# Alginate Improves the Chondrogenic Capacity of 3D PCL Scaffolds In Vitro: A Histological Approach

**DOI:** 10.3390/cimb46040223

**Published:** 2024-04-19

**Authors:** Lara Milián, María Oliver-Ferrándiz, Ignacio Peregrín, María Sancho-Tello, José Javier Martín-de-Llano, Cristina Martínez-Ramos, Carmen Carda, Manuel Mata

**Affiliations:** 1 Department of Pathology, Faculty of Medicine and Dentistry, Universitat de València, Blasco Ibáñez Avenue, 15, 46010 Valencia, Spain; 2 INCLIVA Biomedical Research Institute, Menéndez y Pelayo Street, 4, 46010 Valencia, Spain; 3 IMED Hospital, 46100 Valencia, Spain; 4 Centro de Biomateriales e Ingeniería Tisular (CBIT), Universitat Politècnica de València, Camino de Vera, s/n Ciudad Politécnica de la Innovación, Edificio 8E. Acceso F. Nivel 1, 46022 Valencia, Spain; 5 Biomedical Research Networking Center on Bioengineering, Biomaterials and Nanomedicine (CIBER-BBN), 28029 Madrid, Spain

**Keywords:** PCL, alginate, cartilage regeneration, chondrocyte, tissue engineering

## Abstract

Polycaprolactone (PCL) scaffolds have demonstrated an effectiveness in articular cartilage regeneration due to their biomechanical properties. On the other hand, alginate hydrogels generate a 3D environment with great chondrogenic potential. Our aim is to generate a mixed PCL/alginate scaffold that combines the chondrogenic properties of the two biomaterials. Porous PCL scaffolds were manufactured using a modified salt-leaching method and embedded in a culture medium or alginate in the presence or absence of chondrocytes. The chondrogenic capacity was studied in vitro. Type II collagen and aggrecan were measured by immunofluorescence, cell morphology by F-actin fluorescence staining and gene expression of *COL1A1, COL2A1, ACAN, COL10A1, VEGF, RUNX1* and *SOX6* by reverse transcription polymerase chain reaction (RT-PCR). The biocompatibility of the scaffolds was determined in vivo using athymic nude mice and assessed by histopathological and morphometric analysis. Alginate improved the chondrogenic potential of PCL in vitro by increasing the expression of type II collagen and aggrecan, as well as other markers related to chondrogenesis. All scaffolds showed good biocompatibility in the in vivo model. The presence of cells in the scaffolds induced an increase in vascularization of the PCL/alginate scaffolds. The results presented here reinforce the benefits of the combined use of PCL and alginate for the regeneration of articular cartilage.

## 1. Introduction

Cartilage is a special form of connective tissue capable of resisting compressive forces thanks to its unique tissue architecture. Cartilage plays a relevant role in lining the bone surfaces in the joints, where, in addition to absorbing tensile and compressive forces, it distributes them in a balanced way towards the subchondral plate, protecting the underlying bone tissue [1,2].

Articular cartilage is a form of hyaline cartilage tissue with some peculiarities that include a very defined organization of type II collagen fibers from the surface, where they are organized tangentially to the articular surface, and to the depth, where they are perpendicular to the loads supported by the joint. In addition, like the rest of the varieties of cartilaginous tissue, it is avascular and, particularly, lacks a perichondrium as an adaptation to presenting a smooth surface, which is essential to increasing its cushioning function [3].

The adaptive characteristics of articular cartilage make it a tissue with a minimal regenerative capacity. Its nutrition is produced from the synovial fluid and its regeneration is carried out by the mesenchymal cells included in the underlying bone marrow. This is the basis of certain therapies, including, for example, drilling, abrasion arthroplasty and microfracture [4]. These therapeutic approaches work relatively well for small injuries, but when a lesion of a certain extent occurs, they fail in their regenerative intention. In these cases, a repair tissue consisting of fibrocartilage is generated [5]. For example, the application of osteochondral autograft transfer is restricted to small chondral defects (<2 cm^2^) because of the limitations in graft availability [6]. Osteochondral allograft transplantation is a feasible solution for AC repair due to the avascular nature of cartilage, which alleviates any immunological concerns [7]. Furthermore, the newly formed tissue, which can be considered as a form of transition towards a disorganized dense fibrous connective tissue, is not capable of meeting the strict requirements of the articular cartilage from a biomechanical point of view, leading to a situation of chronic inflammation that, in some cases, can result in total loss of joint function [8].

In this context, articular cartilage engineering has become a priority strategy for regenerative medicine. Different strategies have been used, including autologous chondrocyte transplantation (ACI) or the use of matrices capable of stimulating regeneration from resident cells (MACI) [9]. ACI was introduced in 1987 in Sweden, and in 1994 the first clinical report showing satisfactory results was published [10]. Although ACI has proven to be useful, is not exempt of limitations including that, on the one hand, two surgical processes are needed and, on the other, it is limited to regenerating areas of limited extension in the articular cartilage [11,12]. MACI is based on the use of a biomaterial that acts by maintaining mechanical integrity, withstanding stress loads in the body and thus providing an adequate mechanical environment to cells [13,14].

Hyaline cartilage is also the fundamental structural support tissue of the trachea. It is essential to maintaining the complex biomechanical characteristics of this airway, so any deterioration of its native structure has dramatic consequences in terms of its vital importance as the only way of conducting air to the lungs. Different events that affect the trachea, including malignant neoplasms, benign stenosis secondary to accidental trauma, as well as congenital, inflammatory or iatrogenic conditions, lead to the resection of a circumferential segment of the trachea [15]. In these cases, end-to-end surgical anastomosis of the trachea is the gold standard. However, despite clinical successes, replacing of more than half the length of the trachea remains an unsolved challenge [16]. In these cases, the manufacture of a valid substitute to replace the affected segment of the trachea has been a priority objective in tracheal tissue engineering. Nevertheless, unfortunately these goals remain unreached due to, among many other reasons, the impossibility of achieving adequate regeneration of the hyaline cartilage that forms the structural rings of the trachea [17].

The literature is very extensive in relation to the different biomaterials used in cartilage regeneration. One of the most notable is perhaps polycaprolactone (PCL). PCL is biodegradable, biomechanically compatible with cartilage and has proven its effectiveness in different in vivo models of cartilage regeneration [18,19,20,21,22], as well as in the manufacturing of tracheal patches or rings [23,24].

Chondrogenesis is influenced by the 3D environment in which chondrocytes are cultured. Hydrogels provide the appropriate environment to induce chondrocyte proliferation and differentiation.

Various hydrogel sources have been explored for their potential in cartilage repair, encompassing proteins like collagen, elastin, fibrin and silk fibroin, as well as polysaccharides such as chitosan, chondroitin sulfate and hyaluronic acid [25]. Additionally, algal polysaccharides like alginate, agarose or carrageenan have garnered attention for cartilage regeneration. Alginate, in particular, has drawn interest due to its sulfate groups, which enhance its chemical affinity for mammalian glycosaminoglycans. Moreover, alginate’s minimal interaction with cell integrins aids in preserving the rounded shape of cultured cells, thereby promoting chondrogenesis [26,27]. Alginate hydrogels provide the right environment to induce chondrogenesis. However, their biomechanical properties are not adequate for use in moderate or large cartilage injuries.

A combined scaffold of PCL and alginate could be useful in the regeneration of hyaline cartilage, as, on the one hand, it would allow for adequate support of the maintenance of the biomechanical properties of the injured cartilage and, on the other, it would provide a friendly environment for chondrogenesis. The main objective of this work is, therefore, to generate a mixed scaffold consisting of a porous support of PCL embedded in an alginate hydrogel and to evaluate its chondrogenic properties and biocompatibility. For this, we have generated an in vitro model of chondral differentiation based on research results previously published by our group. Furthermore, we have evaluated the biocompatibility of the scaffolds generated in a subcutaneous implant model carried out in athymic nude mice.

## 2. Materials and Methods

### 2.1. Experimental Design

In this work we have manufactured scaffolds consisting of a porous PCL support embedded in an alginate hydrogel with primary chondrocytes obtained from articular cartilage biopsies. PCL scaffolds were generated and embedded with a cell culture medium or alginate with or without 2 × 10^6^ chondrocytes/mL, previously isolated from human articular cartilage samples. Mixed scaffolds were cultured in a chondrogenic differentiation medium for 4 weeks. We evaluated the expression of collagen type II and aggrecan by means of immunofluorescence, as well as the morphology of cultured chondrocytes by fluorescent staining of F-actin using rhodamine-phalloidin. The relative expression of *COL1A1, COL2A1, COL10, VEGF, RUNX1* and *SOX6* was studied by real time RT-PCR.

The biocompatibility of the scaffolds was evaluated in vivo using a subcutaneous implant model in athymic nude mice. The scaffolds, containing or not-containing alginate and/or chondrocytes, were implanted for 4 weeks into the back of animals. Classical histological stainings (hematoxylin-eosin (HE), Masson’s trichrome) were used to assess biointegration. Vascularization of the scaffolds was studied by immunohistochemical staining for CD31.

### 2.2. PCL Scaffold Manufacture and Characterization

The PCL scaffolds were fabricated by a mixed particle leaching/freeze extraction process, as previously reported [28]. Briefly, using acrylic resin microspheres with sizes of 200 µm (Elvacite 2043, Lucite International, Southampton, UK) by stering at 140 °C to create porogenic templates, PCL (Polysciences Inc., Eppelheim, Germany) was dissolved in dioxane at 20% (*w*/*w*) and injected into templates using a custom-made injection device. Then, molds were immersed in liquid nitrogen in order to force the melted PCL to efficiently fill the pores of the template. Thereafter, the porogen was removed by washing for 3 days in ethanol. Scaffolds samples of 5 mm diameter were stamped out of the disks and irradiated with g-ray irradiation at 25 kGy before use for cell culture.

The PCL frameworks exhibited a porosity ranging from 60–85%, displaying well-connected morphology and uniformity. The pore dimensions matched the diameter of the porogen particles employed, at approximately 200 µm. The elastic modulus of the unfilled, desiccated scaffold was estimated from our earlier research [28] and amounted to roughly 5 MPa.

### 2.3. Mixed PCL-Alginate Scaffolds Manufacture

A 3% alginate solution was prepared as previously reported [25,29]. Briefly, sodium alginate (alginic acid sodium salt derived from brown algae, Sigma-Aldrich, Madrid, Spain) was dissolved while being continuously stirred in a sterile solution (40 mM HEPES and 300 mM NaCl) that had been preheated to 65 °C. After the alginate completely dissolved in the buffer and the solution cooled to room temperature, the pH was regulated to 7.4. The solution was then autoclaved and kept at 4 °C until needed.

Scaffolds were conditioned using a proliferation medium containing Dulbecco’s Modified Eagle Medium (DMEM) supplemented with 10% heat-inactivated fetal bovine serum (FBS), 50 µg/mL ascorbic acid, 1% non-essential amino acids, 1% pyruvate, 100 U penicillin, 100 μg streptomycin and 1% fungizone (Gibco/Thermo Fisher Scientific, Madrid, Spain). To achieve this, 5 mL of a cell culture medium were dispensed into a vacutainer, 3 mm diameter discs were placed in the vacutainer and negative pressure was applied using a 10-mL syringe until the PCL scaffolds sank into the culture medium, at which point they were completely embedded in it. The discs were deposited in a 24-well plate (one disc per well) and, depending on the experimental group, 30 µL of a proliferation medium (see below), with or without chondrocytes or alginate solution (with or without chondrocytes), was dispensed on the top of the discs. In those experimental groups that included chondrocytes, a density of 2 × 10^6^ cells/mL was used. Reticulation of alginate was induced by adding a 10% volume of 102 mM CaCl_2_. Scaffolds were then incubated at 37 °C for 15 min, and 250 µL of an appropriate culture medium was added.

### 2.4. Cell Isolation and Culture

Articular cartilage was sourced from the knee joints of patients undergoing arthroscopy, following previously established protocols [30]. The study adhered to the Declaration of Helsinki and local regulatory requirements and laws. Approval was obtained from the Ethics Committee of the University Clinical Hospital of Valencia (Spain, identification code 2016/27, approved in April 2019).

The harvested tissue was cleansed with DMEM supplemented with 100 U penicillin, 100 μg streptomycin and 0.4% fungizone (Gibco/Thermo Fisher Scientific, Madrid, Spain). Subsequently, cartilage fragments in supplemented DMEM were diced and subjected to enzymatic digestion using the following protocol: (i) Incubation with 0.5 mg/mL hyaluronidase (Sigma-Aldrich, St. Louis, MO, USA) in a shaking water bath at 37 °C for 30 min and (ii) removal of the hyaluronidase solution followed by the addition of 1 mg/mL pronase (VWR International, Barcelona, Spain). Incubation continued in a shaking water bath at 37 °C for 60 min, (iii) and washing of cartilage pieces with supplemented DMEM and (iv) addition of 0.5 mg/mL collagenase-IA (Sigma-Aldrich, Madrid, Spain) and overnight digestion in a shaking water bath at 37 °C followed. The resultant cell suspension was filtered through a 70-µm pore nylon filter (BD Biosciences, San Jose, CA, USA) to eliminate tissue debris. The cells were then centrifuged, and the cell pellet was washed with DMEM supplemented with 10% inactivated FBS. Finally, the isolated cells were cultured under standard conditions (37 °C, 5% CO_2_, 95% air humidified atmosphere) in a proliferation medium consisting of DMEM supplemented with 10% heat-inactivated FBS, 50 µg/mL ascorbic acid, 1% non-essential amino acids, 1% pyruvate, 100 U penicillin, 100 μg streptomycin and 1% fungizone.

Chondral differentiation was initiated by incubating expanded chondrocytes in a differentiation medium comprised of DMEM supplemented with 1% insulin-transferrin-sodium selenite medium supplement (BD Biosciences, Madrid, Spain), 50 µg/mL ascorbic acid, 10 ng/mL TGF-β1 and 1% heat-inactivated FBS.

### 2.5. Immunofluorescence Staining of Type II Collagen and Aggrecan

The expression levels of type II collagen (COLII) and aggrecan (ACAN) were assessed in a cell culture using specific antibodies obtained from Sigma-Aldrich, Madrid, Spain, as previously documented [31]. Scaffolds containing cells were transferred onto 8-well cell culture slides (Millicel, Sigma-Aldrich, Madrid, Spain). The cells were fixed with 4% paraformaldehyde in PBS (pH 7.4) for 10 min, then washed with PBS, and permeabilized with 0.1% Triton X-100 in PBS for 5 min. Following three washes, they were incubated for 30 min with a blocking solution (1% bovine serum albumin [BSA] and 1.1% Tween-20 in PBS). Subsequently, the scaffolds were incubated overnight at 4 °C with the appropriate primary antibodies (diluted in antibody diluent solution at 1:100 for ACAN and 1:500 for COLII antibody). After three additional washes, the scaffolds were incubated with the following corresponding secondary antibodies: an anti-mouse (for COLI and COLII) or anti-rabbit (for ACAN) FITC-conjugated antibody (Sigma-Aldrich, Madrid, Spain), diluted at 1:200. Following the final washes, the nuclei were stained with 4′,6-diamidino-2-phenylindole (DAPI), and the samples were examined using a Leica DM2500 fluorescence microscope (Leica, Wetzlar, Germany)

### 2.6. Fluorescence Staining of F-Actin

To assess F-actin, rhodamine-conjugated phalloidin (Molecular Probes, Thermo Fisher Scientific, Madrid, Spain) was employed. The scaffolds were positioned in 8-well cell culture slides and pre-treated with PBS containing 1% BSA for 20–30 min to minimize non-specific background staining. Following this, each sample was stained for 20 min with 5 µL of rhodamine-conjugated phalloidin methanol stock solution, diluted in 200 µL of PBS. After multiple rinses with PBS, the nuclei were counterstained with DAPI and observed under a DM2500 fluorescence microscope.

### 2.7. Relative Gene Expression Analysis

Total RNA was extracted from cell-containing scaffolds using TRIzol reagent (Thermo Fischer Scientific Inc., Waltham, MA, USA) according to the manufacturer’s instructions. RNA concentration was determined using a Nanodrop 2000 spectrophotometer (Fischer Scientific, Madrid, Spain). Only extracts with a ratio 260/280 > 1.8 were used. RNA integrity (RI) was evaluated by capillary electrophoresis using a Bioanalyzer (Agilent Technologies, Santa Clara, CA, USA). For the determination of gene expression levels, only extracts with an RI number (RIN) close to 10 were used.

Random hexamers were used to synthesize complementary DNA (cDNA) using TaqMan RT reagents (Applied Biosystems, Foster City, CA, USA) following the manufacturer’s instructions. Gene expression levels were assayed by reverse transcriptase polymerase chain reaction (RT-PCR) using on Demand Assays (Applied Biosystems, Madrid, Spain), as described previously [31]. The reactions were carried out in a 7900HT real-time Thermocycler (Applied Biosystems, Madrid, Spain). The comparative ΔΔCt method with glyceraldehyde 3-phosphate dehydrogenase (*GAPDH*) as an endogenous control was used to calculate relative gene expression levels, which expresses the fold-variation in the experimental group with respect to the control group [31].

Total RNA was isolated from cell-laden scaffolds utilizing TRIzol reagent (Thermo Fischer Scientific Inc., Waltham, MA, USA) following the manufacturer’s protocol. RNA concentration was quantified using a Nanodrop 2000 spectrophotometer (Fischer Scientific, Madrid, Spain), and only samples with a 260/280 ratio exceeding 1.8 were included. RNA integrity (RI) was assessed via capillary electrophoresis using a Bioanalyzer (Agilent Technologies, Santa Clara, CA, USA), with samples featuring an RI number (RIN) close to 10 selected for further analysis.

Complementary DNA (cDNA) synthesis was performed using random hexamers and TaqMan RT reagents (Applied Biosystems, Foster City, CA, USA) as per the manufacturer’s instructions. Gene expression levels were evaluated by reverse transcriptase polymerase chain reaction (RT-PCR) utilizing Assays On Demand (Applied Biosystems, Madrid, Spain), following established procedures [31]. The reactions were conducted in a 7900HT real-time Thermocycler (Applied Biosystems, Madrid, Spain).

Relative gene expression levels were determined using the comparative ΔΔCt method with glyceraldehyde 3-phosphate dehydrogenase (GAPDH) as an endogenous control. This approach quantifies the fold-change in gene expression in the experimental group relative to the control group [31].

### 2.8. In Vivo Study

The procedures involving experimental animals were conducted in accordance with previously established protocols [32]. The animal study protocol received approval from the Ethics Committee of Animal Experimentation (CEEA) of the University of Valencia (Spain) under protocol code 2019/VSC/PEA/0146 (2019). Male athymic nude mice, approximately 8 weeks old and weighing between 20 and 25 g, were obtained from Charles River Laboratories, Inc., Wilmington, MA, USA, for use in this study. The sample size was calculated using a F test-ANOVA of repeated measures assuming a significance level of 0.05, a power of 0.8 and a minimum effect size to detect of 0.5.

**Figure 1:**
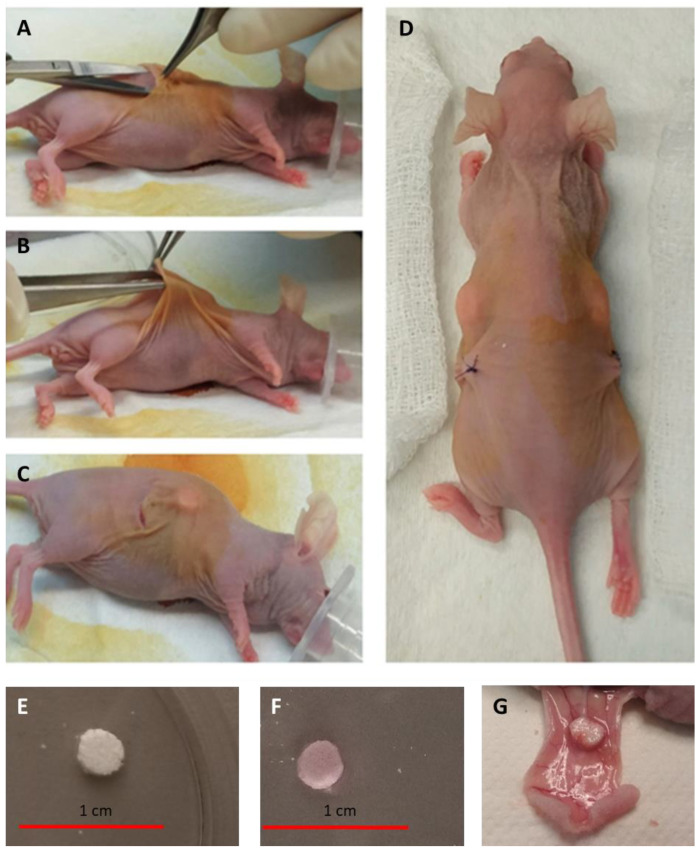
Detail of the PCL scaffold implantation in the in vivo model, nude athymic mice. A midline incision through the dorsal skin was created (**A**) and two subcutaneous pockets were formed by blunt dissection (**B**). A single scaffold was placed within each pocket (**C**). The dorsal incision was closed (**D**). Representative pictures of dry (**E**) and medium-embedded scaffolds (**F**) prior to implant are shown. Panel (**G**) shows a representative picture of a scaffold 4 weeks post-implantation.

Following surgery, the animals received ibuprofen (50–80 mg/kg) via drinking water for 2 days. A total of 8 animals were included in the study. Among these, 4 were implanted with discs containing a culture medium, while the remaining 4 received discs embedded in alginate. Control discs without cells were implanted in the left pockets, while discs containing cells were implanted in the right pockets. Representative illustrations of the surgical procedure can be seen in Figure 1.

### 2.9. Histological Evaluation of Implanted Scaffolds

Four weeks post-implantation, the mice were euthanized, and the scaffolds were subjected to standard histological assessments. Initially, the scaffolds were rinsed with PBS and then fixed with 4% formaldehyde at room temperature for 1 h. Subsequently, each scaffold was halved, and each half was individually processed and embedded in paraffin. From these paraffin blocks, 5-μm thick sections were obtained. These sections were then stained using Masson’s trichrome staining technique. The stained sections were examined under a bright-field microscope (DM 4000B; Leica Biosystems, Barcelona, Spain), and images were captured using a Leica DFC420 digital camera [30]. This allowed for the analysis of the tissue morphology and the evaluation of scaffold integration and tissue response.

To assess CD31 expression, immunohistochemistry was conducted using a specific mouse anti-human antibody (D8V9E, Cell Signaling Inc., Danvers, MA, USA) at a 1:100 dilution. Initially, sections were deparaffinized and rehydrated using graded ethanol solutions, followed by rinsing in distilled water. To block endogenous peroxidase activity and nonspecific binding, sections were treated with 0.3% H_2_O_2_ and 10% normal horse serum, respectively. Antigen retrieval was achieved through proteinase K incubation for 10 min. The Dako EnVision amplification system (Cytomation EnVision System-labelled polymer-HRP anti-mouse, Agilent, Santa Clara, CA, USA) was utilized, followed by chromogenic development using 3,3′-diaminobenzidine (Dako, Barcelona, Spain) as per the manufacturer’s instructions, resulting in brown-staining of immunoreactive structures. Finally, sections were counterstained with Mayer’s hematoxylin (Sigma-Aldrich, Madrid, Spain) to visualize nuclei [33]. This comprehensive staining protocol facilitated the examination and analysis of CD31 expression within the tissue sections.

### 2.10. Data Presentation and Analysis

For in vitro experiments, all determinations were carried out in triplicate. Cell density in the scaffolds was measured in the immunofluorescence preparations using the Image-Pro plus 7 software (Media Cybernetics, Rockville, MD, USA) according to DAPI staining, as previously reported [25]. At least ten different fields from each sample were double-blind evaluated.

In vivo studies were used utilizing 4 animals for each of the experimental groups included. For microscopy experiments, representative images of 5 fields for each stain were considered. Data are presented as the mean ± SD. Statistical analysis was carried out using an analysis of variance (ANOVA) followed by a Tukey’s multiple-comparison test (GraphPrim Version 5, GraphPad Software Inc., San Diego, CA, USA). Significance was accepted at *p* < 0.05.

## 3. Results

### 3.1. Alginate Improves Chondrogenic Properties of PCL Scaffolds

**Figure 2:**
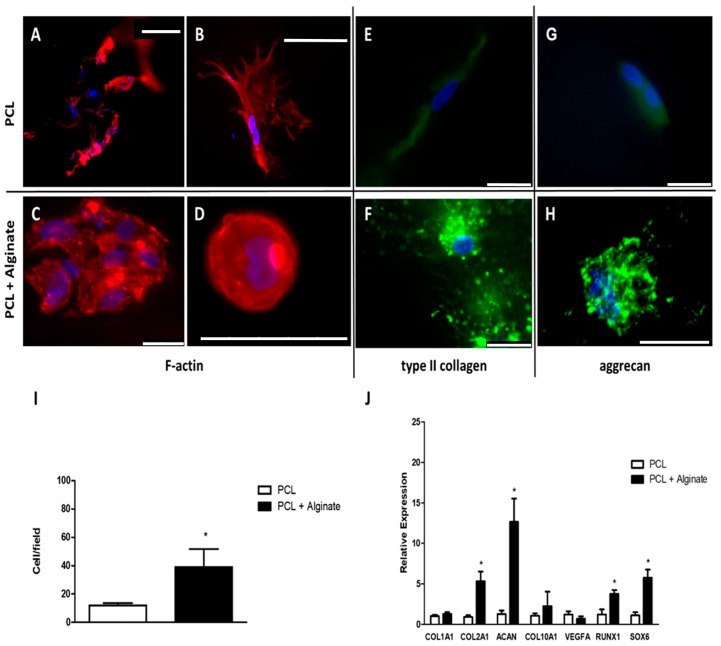
Alginate improves chondrogenic properties of PCL scaffolds in vitro. Human primary chondrocytes were cultured in PCL scaffolds embedded in culture medium (**A**,**B**,**E**,**G**) or alginate (**C**,**D**,**F**,**H**) and cultured in chondrogenic medium for 4 weeks. F-actin fibers were stained using rhodamine-conjugated phalloidin (**A**–**D**). Panel (**A**,**C**) show panoramic view, while panels (**B**,**D**) show detailed images. Type II collagen and aggrecan expression was studied by immunofluorescence ((**E**,**F**) and (**G**,**H**), respectively). The number of cells was estimated by counting nuclei in DAPI stained samples (**I**). RT-PCR was used to estimate the relative gene expression changes in COL1A1, COL2A1, ACAN, COL10A1, VEF, RUNX1 and SOX6 (**J**). The results are representative of *n* = 3 independent experiments. For cell counts, five fields of each replicate were considered. Mean ± SD is represented in panels (**I**,**J**). * *p* < 0.05 versus control group (chondrocytes cultured in PCL scaffolds without alginate). Scale bar (white bar) equals to 75 (**A**,**B**) or 25 (**C**–**H**) µm.

First, we aimed to study whether the alginate improved the chondrogenic properties of the PCL scaffolds. To achieve this, we cultured chondrocytes in PCL scaffolds with or without alginate for 4 weeks in a chondrogenic differentiation medium. Initially, chondrocytes were seeded in the scaffolds at a density of 2 × 10^6^ cells/mL. Due to the hydrophobic nature of the PCL scaffolds, they were previously embedded in a culture medium, applying negative pressure, as described in the methodology section. After the culture time, scaffolds were processed and a histological study was carried out in relation to the morphology and disposition of the cells in the scaffolds (by means of fluorescent F-actin staining), expression of type II collagen and aggrecan (by immunofluorescent staining) and changes in the gene expression (determined by real time RT-PCR) of well-known chondrogenic markers. Representative results are shown in Figure 2.

Chondrocytes cultured in PCL scaffolds grown attached to the surfaces of the trabeculae (Figure 2A). These cells acquired a spindle-shaped morphology with abundant processes, and F-actin fibers were also observed (Figure 2B). Alginate produced apparent changes in both the distribution and morphology of cultured chondrocytes. On the one hand, the chondrocytes proliferated inside the PCL trabeculae and did not adher to the scaffold walls, and, although individual cells could be observed, they tended to clump together in large spheroids (Figure 2C). These cells acquired a round shape with few F-actin fibers preferentially distributed at the periphery of the cell (Figure 2D). These changes were correlated with a notable increase in the expression of type II collagen and aggrecan with respect to cells cultured in PCL discs without alginate, detecting intra and intercellular deposits of these proteins (Figure 2E–H). Changes were not only detected in relation to the morphology or secretory profile of the chondrocytes cultured in the scaffolds, but also in relation to cell density, which significantly increased in the alginate PCL discs compared to the control group (Figure 2I).

For a better comparison of the chondrogenic capacity of the scaffolds used, relative gene expression changes in key-genes involved in chondrogenesis were studied. Results are summarized in Figure 2J. Gene expression levels of chondrocytes cultured in PCL scaffolds without alginate were used as a control. *GAPDH* expression was used as a house-keeping reference gene. Regarding *COL1A1*, we found low levels of expression in both experimental groups, with a Ct close to 37 in each. No changes were found when comparing cells cultured in both scaffold types. Expression of *COL2A1* and *ACAN* was detected in all the samples analyzed. Chondrocytes cultured in PCL scaffolds with alginate showed significantly higher levels of both proteins in comparison with the control group. Concerning *COL10A1* and *VEGFA*, a similar trend than that for *COL1A1* was observed, finding low levels of expression in all samples without significant changes between groups. Finally, a significant increase in the relative expression of RUNX1 and SOX6 in the samples of chondrocytes cultured on PCL discs with alginate was found.

### 3.2. Biocompatibility of PCL Scaffolds In Vivo

**Figure 3:**
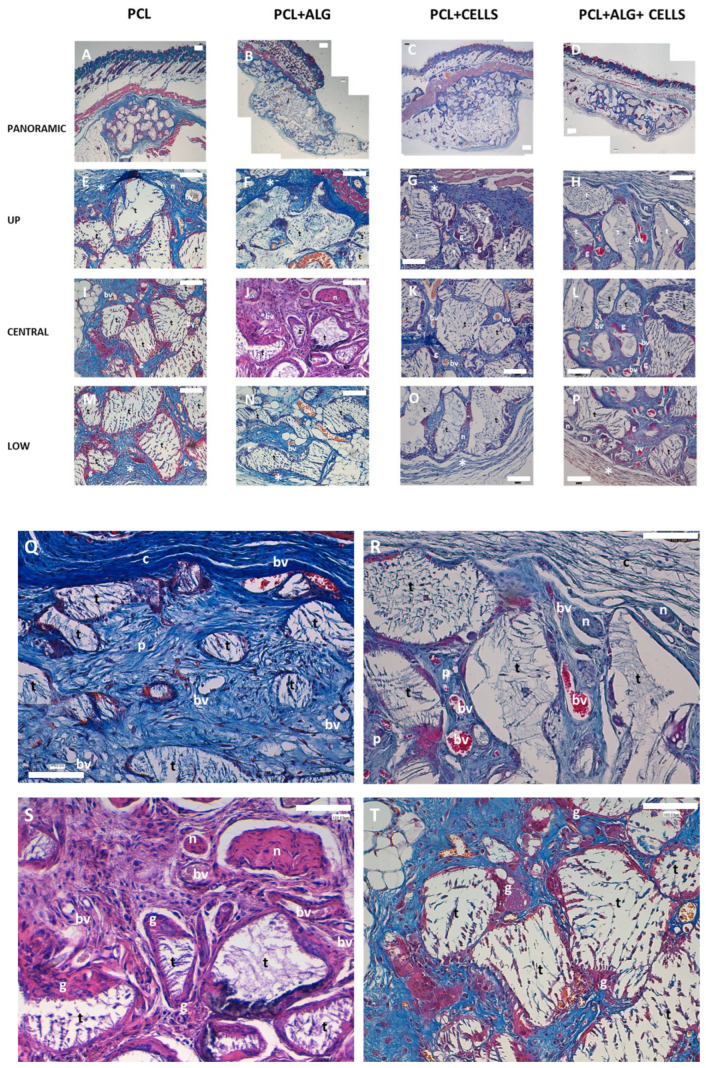
Biocompatibility of mixed PCL/alginate scaffolds. PCL scaffolds were manufactured and embedded with culture medium without (**A**,**E**,**I**,**M**) or with (**C**,**G**,**K**,**O**) cells or alginate without (**B**,**F**,**J**,**N**) or with (**D**,**H**,**L**,**P**) cells. The scaffolds were implanted subcutaneously in the back of athymic nude mice. The samples were histological processed and Masson’s trichrome or HE stained. Representative images of *n* = 4 samples are shown. * (capsule), bv (blood vessel), n (nerve fibers), g (giant cells), t (trabeculae). Details of representative findings are shown in panels (**Q**–**T**) (PCL disks with cells and alginate): * (capsule), bv (blood vessel), n (nerve fibers), g (giant cells), t (trabeculae), p (porous), c (fibrous capsule). Scale bar (white bar) equals to 100 µm.

We studied the biocompatibility of the manufactured scaffolds using an animal experimental model developed in athymic nude mice. Discs with or without alginate, containing or not-containing cells, were implanted in the hypodermis of the back of mice as described in the Section 2. The animals were sacrificed, and histological analysis of samples was undertaken to study the biocompatibility of the scaffolds. The histological study was carried out in the upper (the side of the disk closest to the surface), the central and the lower (the side farther from the surface) portion of the scaffolds. Representative results are shown in Figure 3A–P, and details of the observed structures are shown in Figure 3Q–T. All discs analyzed were surrounded by a fibrous capsule (Figure 3A–D). These capsules were composed of a dense connective tissue with thick bundles of non-organized collagenous fibers (Figure 3E–H, see * label, and Figure 3Q). Large blood vessels and nerve fibers were observed inside the capsules (Figure 3, bv and n labels, details in panel R). Inside the porous, a well-organized loose connective tissue was observed with small blood vessels and, in some locations, thin bundles of nerve fibers (Figure 3C,D,S). Characteristic stromal cells surrounded by collagen fibers were observed (Figure 3Q–T, p labelled). No reactive inflammatory response was found in any of the animals.

Macrophages and giant cells were found surrounding the trabeculae surface of the scaffold (Figure 3, g label, details in panel T, g label). A partial degradation of the PCL was observed, and a characteristic immature loose connective tissue was found inside the trabecular with thin collagen fibers and stromal cells (Figure 3, t label). No blood vessels or nerves were found inside the trabeculae. No differences between the different groups analyzed were found, with all of them showing a good biocompatibility.

**Figure 4:**
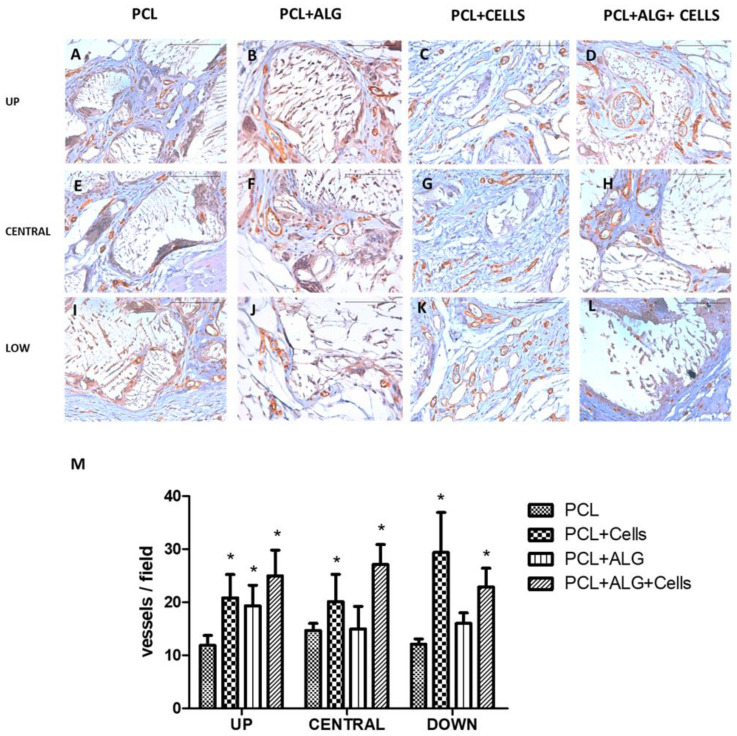
Vascularization analysis of mixed PCL/alginate scaffolds. PCL scaffolds were manufactured and embedded with culture medium without (**A**,**E**,**I**) or with (**C**,**G**,**K**) cells or alginate without (**B**,**F**,**J**) or with (**D**,**H**,**L**) cells. Then, scaffolds were implanted subcutaneously in the back of athymic nude mice. CD31 was studied by immunohistochemistry. A morphometric analysis of stained blood vessels was conducted (**M**). Representative results of *n* = 4 samples are shown. * *p* < 0.05 compared to control group (PCL without alginate and without cells). Scale bar (white bar) equals to 100 µm.

Finally, we aimed to analyze vascularization of implanted scaffolds. To achieve this, CD31 was immunohistochemically detected and a morphometric analysis of the vessel was conducted. Results are represented in Figure 4. A well-developed blood vessels network was neo-formed inside the scaffolds. We found large as well as small blood vessels in the upper side of the scaffolds, which developed through the pores of the PCL scaffold, occupying the entire internal part of the discs. In the central portion and in the lower side of the scaffolds, blood vessels were smaller in caliber than in the superficial portion. Regarding the morphometric analysis, in all the groups analyzed, the number of blood vessels was greater in those scaffolds containing cells. Regarding alginate, we found statistically significant differences in the number of vessels compared to the scaffolds that contained only PCL, but only on the upper side of the scaffolds.

## 4. Discussion

The main objective of this work is to evaluate if, on the one hand, the combination of alginate with a porous PCL scaffold increases the chondrogenic properties of this scaffold and if, on the other hand, it influences the biointegration of said scaffolds. For the first objective, we have used an in vitro model consisting of the culture of chondrocytes in manufactured scaffolds embedded or not-embedded in alginate. For the second, we have used an in vivo model developed in athymic nude mice. Our results indicate that, on the one hand, the use of alginate improves the chondrogenic properties of PCL, and, on the other hand, it does not affect the biointegration of the biomaterial.

The main function of hyaline cartilaginous tissue is to be a support tissue for compressive forces, so that, in the joint, it protects the subchondral bone from the mechanical stress generated by joint movement, while in other organs, such as the trachea, it prevents collapse, therefore being vital for the flow of air into the lung territory [34]. The peculiar characteristics of hyaline cartilage derive from the complex structure of its extracellular matrix, generated and maintained by chondrocytes. When this structure is damaged for different reasons, the fibrous repair tissue that replaces it lacks the ability to resist compression forces, with dramatic effects that can lead to joint failure or, in the case of tracheal stenosis, death.

Thus, the repair of hyaline cartilage generates a fibrotic tissue with abundant type I collagen that is inadequate for the re-establishment of the functions of the affected organs, which is why the regeneration of this tissue has become a priority objective of tissue engineering. Regarding articular cartilage, there are thousands of works with different approaches for its regeneration, the results of which, unfortunately, have not satisfactorily resolved the problem of the regeneration of this tissue. This particular variety of articular hyaline cartilage presents an extracellular matrix with a complex organization in layers or zones with different arrangements and characteristics of type II collagen fibrils, which allows for correct distribution and dissipation of compression and friction forces. In this tissue, it is essential not only to achieve regeneration of the hyaline tissue, but also to induce remodeling by the chondrocytes to generate a sufficiently organized tissue. Otherwise, even if initial regeneration of the altered cartilage is achieved, the newly formed tissue will end up failing in its cushioning role. This is one of the causes of the failure of strategies based on the use of cells isolated or embedded in hydrogels. For this reason, different biomaterials have been tested that ideally should serve as a first mechanical support and then be replaced by newly formed hyaline cartilage. Thus, biomaterials such as PCL have demonstrated their usefulness, as, on the one hand, they can be manufactured with an elastic modulus similar to that of native cartilage and, on the other hand, are biodegradable and therefore susceptible to being remodeled and replaced by normal neoformed cartilage tissue. Concerning airways, although the biomechanical requirements are not as complex as in the case of articular cartilage, it is evident that the use of biomaterials that support the structure and prevent its collapse is critical. Furthermore, while articular cartilage is nourished directly from the synovial fluid, tracheal cartilage does so through an intricate network of small blood vessels that irrigate each of the tracheal rings from the branches of the mammary arteries, so any tissue replacement must, in addition to generating a minimum support, be vascularized from the adjacent tissues.

For these reasons, as a reinforcement of our scaffold, we have chosen PCL, as, on the one hand, the manufacturing process used generates a regular porous structure with appropriate biomechanical characteristics and, on the other hand, it is a chondrogenic material, as has already been demonstrated in vitro and in models of articular cartilage regeneration. The manufactured scaffolds present a regular interconnected network of pores measuring 200 μm on average, which is an optimal size for bone or cartilage regeneration models, and that show an elastic modulus of 5 MPa, which is in the range of normal hyaline cartilage [35]. Thus, the manufactured PCL scaffolds meet the structural and biomechanical criteria necessary to be used as a support element for cartilage tissue [36,37].

The regeneration of cartilaginous tissue requires an influx of chondral precursors that must differentiate into chondrocytes. The faster this occurs, the more successful the regeneration will be, so it is important to improve the chondrogenic properties of the biomaterials used in cartilaginous tissue engineering to maximize the regenerative potential of the scaffolds used. Alginate hydrogels have demonstrated excellent chondrogenic properties both in vitro and in vivo. This is because, on the one hand, they maintain certain similarities with the chondral matrix and, on the other hand, they lack elements that bind to the chondrocytes, so these cells do not adhere to any component, which favors their differentiation process toward mature secretory chondrocytes. This process is characterized by a change in the cell shape, which acquired a round morphology, and by an increase in the expression of characteristic cartilage matrix proteins, such as type II collagen and aggrecan.

For all these reasons, we proposed a combination of both biomaterials to generate a mixed scaffold that would allow basic structural support as well as generate an adequate chondrogenic environment. To evaluate this, we cultured human chondrocytes from articular cartilage samples and suspended them in an alginate solution following a model widely used by our research group. We embedded the PCL scaffolds with this suspension and induced the reticulation of the alginate. After culturing the scaffolds for 4 weeks with a chondrogenic culture medium, we compared them with those scaffolds in which we cultured chondrocytes without alginate. We observed that, in the absence of alginate, chondrocytes adhered to the PCL trabeculae and grew, acquiring an elongated morphology, in concordance with results obtained by other researchers [38]. Alginate significantly improved the chondrogenic properties of the PCL scaffolds. On the one hand, the chondrocytes showed a homogeneous distribution, remaining suspended in the pores of the scaffold assuming a spherical morphology with an F-actin organization pattern similar to that of native chondrocytes. These cells showed clusters, often large, of chondrocytes, suggesting greater proliferation of cells cultured within the mixed scaffold compared to PCL scaffolds lacking alginate. To verify this, we performed a morphometric analysis within the analyzed scaffolds. Although this is not a measure of proliferation, it allowed us to conclude that the number of cells within alginate-containing scaffolds was significantly greater than in the scaffolds that did not contain alginate, indicating greater cellularity in the first. These changes were also correlated with a higher protein expression of type II collagen and aggrecan, two of the most characteristic elements of the chondral matrix, further supporting our hypothesis. These proteins were expressed in the PCL scaffolds, as described previously, but their expression significantly increased in the mixed PCL/Alginate scaffolds at both the protein and mRNA levels. *COL1A1* is one of the fundamental genes that differentiate the fibrous cartilage matrix from the standard hyaline cartilage matrix, being, therefore, an important marker of fibrosis. We have found low levels of expression of this gene in all the experimental groups analyzed, without also finding significant differences because of the incorporation of alginate to the scaffold, which supports the results of previous studies supporting the chondrogenic properties of this type of scaffolds. We also measured the expression of genes relevant to the chondrogenesis process. We evaluated the expression of two known hypertrophy markers such as *COL10A1* and *VEGF*. *COL10A1* is a well-known marker of chondrocyte hypertrophy, while *VEGF* induces angiogenesis and characteristic events of the degradation of articular cartilage in pathologies such as osteoarthritis. Once again, we found low levels of expression of these markers in all the groups analyzed, reinforcing the chondrogenic potential of the scaffolds used. We found, however, significant differences in the expression of *RUNX1* and *SOX6*. *RUNX1* has been reported to be widely expressed in chondrocyte progenitor cells and to stimulate chondrogenesis [39,40]. Furthermore, *RUNX1* contributes to the production of the chondral matrix by enhancing type II collagen expression in cooperation with *SOX* proteins [41]. In accordance with this observation, we found a significant overexpression of both *RUNX1* and *SOX6* when alginate was incorporated to the PCL scaffolds, which supports the results observed in relation to the increase in the expression of *COL2A1* and *ACAN*, as well as in the immunofluorescence detection of the proteins codified by these genes.

Next, we decided to evaluate in vivo the biocompatibility of the scaffolds. To achieve this, we use a standardized model generated in athymic nude mice. We used this model because we wanted to evaluate the effect of scaffolds containing human chondrocytes, avoiding the use of immunosuppressant, and because it is a model widely used in the literature. The results demonstrated an excellent biocompatibility of all the scaffolds used. A dense connective tissue capsule developed surrounding the PCL discs implanted in the mice. This capsule contained blood vessels and nerves that invaded and branched through the interior of the scaffold, generating a vital network through it. The blood vessels found in the internal and lower part of the scaffolds suggest that they branched from the superficial side of the implants, which could indicate an organized colonization of the scaffolds. We found that the pores of the PCL discs were invaded by a connective tissue that was looser than that of the capsule, and the tissue formed de novo was well organized in relation to the distribution of collagen fibers, stromal cells, blood vessels and nerves. Furthermore, we observed that PCL trabeculae were being partially degraded by macrophagic cells, which delimited the entire surface of the trabeculae in the form of giant cells and that a primitive connective tissue was beginning to form with some unorganized collagen fibers and abundant fibroblastic cells.

Of the parameters analyzed in this histological study, we only found significant differences in relation to vascularization. We found a greater number of blood vessels in those experimental groups that included cells. This is consistent with multiple studies that demonstrate greater vascularization of scaffolds that contain cells compared to acellular ones. It is important to highlight that the in vivo model used is not a cartilage regeneration model, but rather a biocompatibility model. It is expected that the implantation of these scaffolds in articular cartilage will have a different behavior, due to the elements that characterize the microenvironment in said tissue. The study of the behavior of these scaffolds in an injured joint cartilage remains pending and is a future objective to be developed by our research groups, with demonstrated experience in this field.

The organization of the connective tissue around the implanted scaffolds and the good vascularization found could support the use of these scaffolds in other models, such as, for example, for the regeneration of tracheae, where vascularization is the main limitation for the use of bioengineered scaffolds. The good integration of the scaffolds used would allow their use in vivo prevascularization for a subsequent implantation in the airway, constituting a potential scaffold for the regeneration of the airway in diseases that, to date, have not yet been resolved, as is the case of tracheal stenosis.

In summary, the results presented herein indicate that the addition of alginate to porous PCL scaffolds substantially improves their chondrogenic properties, showing excellent biocompatibility in vivo. In this way, we can conclude that the combination of alginate and PCL porous scaffolds could be, therefore, a potentially suitable scaffold for use in the regeneration not only of articular cartilage, but of other types of cartilage, such as tracheal cartilage, as well.

## Data Availability

Data is contained within the article.
